# BCRInsight: an antibody language model to decode biological signals from BCR sequences

**DOI:** 10.1093/bib/bbag154

**Published:** 2026-04-14

**Authors:** Hailong Zhao, Shang Lou, Xuhua Li, Yiyang Gao, Wenjing Cao, Hongcang Gu, Fan Zhang

**Affiliations:** Anhui Province Key Laboratory of Medical Physics and Technology, Institute of Health and Medical Technology, Hefei Institutes of Physical Science, Chinese Academy of Sciences, Hefei, Anhui Province 230031, China; Science Island Branch, Graduate School of USTC, University of Science and Technology of China, Hefei, Anhui Province 230026, China; Anhui Province Key Laboratory of Medical Physics and Technology, Institute of Health and Medical Technology, Hefei Institutes of Physical Science, Chinese Academy of Sciences, Hefei, Anhui Province 230031, China; Science Island Branch, Graduate School of USTC, University of Science and Technology of China, Hefei, Anhui Province 230026, China; Anhui Province Key Laboratory of Medical Physics and Technology, Institute of Health and Medical Technology, Hefei Institutes of Physical Science, Chinese Academy of Sciences, Hefei, Anhui Province 230031, China; Science Island Branch, Graduate School of USTC, University of Science and Technology of China, Hefei, Anhui Province 230026, China; HIT Center for Life Sciences, School of Life Science and Technology, Harbin Institute of Technology, Harbin, Heilongjiang Province 150080, China; Anhui Province Key Laboratory of Medical Physics and Technology, Institute of Health and Medical Technology, Hefei Institutes of Physical Science, Chinese Academy of Sciences, Hefei, Anhui Province 230031, China; Science Island Branch, Graduate School of USTC, University of Science and Technology of China, Hefei, Anhui Province 230026, China; Anhui Province Key Laboratory of Medical Physics and Technology, Institute of Health and Medical Technology, Hefei Institutes of Physical Science, Chinese Academy of Sciences, Hefei, Anhui Province 230031, China; Science Island Branch, Graduate School of USTC, University of Science and Technology of China, Hefei, Anhui Province 230026, China; Anhui Province Key Laboratory of Medical Physics and Technology, Institute of Health and Medical Technology, Hefei Institutes of Physical Science, Chinese Academy of Sciences, Hefei, Anhui Province 230031, China; Science Island Branch, Graduate School of USTC, University of Science and Technology of China, Hefei, Anhui Province 230026, China

**Keywords:** B-cell receptor, antibody language model, contrastive learning

## Abstract

The B-cell receptor (BCR) repertoire encodes not only antigen-binding specificity but also intrinsic signatures reflecting B-cell functional states and differentiation trajectories. Deciphering the intricate sequence semantics embedded within these repertoires is pivotal for elucidating immune dynamics and expediting antibody discovery. Although single-cell sequencing provides high-resolution insights, its scalability and cost remain major obstacles, leaving population-level repertoire data underexploited. Furthermore, conventional bioinformatics approaches struggle to model the high-order, non-linear semantic dependencies inherent in antibody sequences. To address these challenges, we present BCRInsight, an antibody-specific pretrained language model that integrates a Transformer architecture with phenotype-aware contrastive learning. Pretrained on 80 million human BCR sequences, BCRInsight learns biologically meaningful contextual representations that encode subtle signatures of B-cell activation, maturation, and clonal evolution. Extensive benchmarking demonstrates that BCRInsight achieves state-of-the-art performance across multiple downstream tasks, particularly in paratope prediction. Further evaluation on diverse single-cell immune cohorts, including healthy, neoplastic, and viral infection states, reveals cross-scenario robustness and superior generalization relative to existing methods. Notably, attention-based analyses show that high-attention regions correspond closely to physical antigen-contact residues, highlighting emergent structural interpretability derived solely from self-supervised learning. Collectively, BCRInsight establishes a new paradigm for decoding the “language” of antibodies, offering a scalable and interpretable framework for computational immunology and rational antibody engineering.

## Introduction

The adaptive immune system relies on the immense diversity of B-cell receptors (BCRs) to recognize a vast and ever-evolving landscape of pathogens [1, 2]. Through somatic recombination of V(D)J gene segments followed by somatic hypermutation (SHM), the human immune repertoire can theoretically generate over 10^13^ unique BCR sequences [3]. Beyond conferring antigen-binding specificity, these sequences harbor intrinsic molecular motifs, often referred to as “fingerprints”, that encode B-cell developmental trajectories and functional states, such as the differentiation from naive to memory phenotypes [4]. However, accurately decoding these high-dimensional “functional semantics” from massive, sparse, and noise-prone BCR sequencing data remains a major challenge in computational immunology.

Traditional methods predominantly rely on aggregate statistics such as V(D)J gene usage frequency, Complementarity-determining region 3 (CDR3) length distribution, or somatic mutation rate [5–7]. Although these metrics provide a macroscopic view of repertoire composition, they fail to capture the complex, non-linear amino acid dependencies within individual sequences. To enrich representation, engineered features such as k-mer frequencies and physicochemical property encoding have been introduced [8–13]. Yet, these hand-crafted representations depend heavily on prior knowledge and often overlook long-range residue interactions, thereby limiting performance in tasks requiring fine-grained structural or functional resolution, such as antibody paratope prediction or B-cell subtype classification.

Recently, pretrained language models (PLMs) based on Transformer architecture [14] have transformed protein sequence analysis. By treating proteins as the “language of life”, PLMs learn the syntactic and semantic rules of amino acid sequences through large-scale unsupervised training [15]. Emerging evidence suggests that antibody-specific PLMs, trained specifically on BCR repertoires, can more precisely capture evolutionary constraints and immune-specific semantics than general protein language models like ESM or ProtBERT [15–20]. This has motivated the development of domain-tailored architectures dedicated to modeling the unique characteristics of BCR sequences.

Despite these advances, existing antibody-specific PLMs exhibit notable limitations. For instance, AntiBERTa [21] effectively encodes structural properties via masked language modeling (MLM) [22] but lacks explicit integration of biological context, restricting its capacity to resolve the subtle biological differences among B-cell subtypes. Similarly, Sapiens [23], although useful for humanization assessment, employs a relatively shallow four-layer architecture that limits representation depth [24]. More crucially, current models largely overlook the rich supervisory signals embedded in repertoire metadata, such as isotype or tissue origin. Without strategies like phenotype-aware contrastive learning (CL) [25] to align biologically similar sequences within the embedding space, these models struggle to achieve optimal discriminative power in downstream phenotype prediction tasks.

To address these challenges, we present BCRInsight, a 12-layer antibody-specific PLM model trained on 80 million human BCR sequences. In contrast to methods relying solely on unsupervised masked modeling, BCRInsight incorporates phenotype-aware CL [26]. By leveraging weak supervision signals derived from metadata (e.g. cell subtype) to cluster biologically related sequences within the latent representation space, this design enables explicit modeling of functional semantics associated with B-cell activation, maturation, and clonal evolution.

The resulting representations yield high biological fidelity and robust generalization, making BCRInsight particularly valuable for immune repertoire analysis. Notably, the model can accurately deconvolute subpopulation composition from bulk BCR sequencing data [27], offering a cost-effective alternative to single-cell RNA/BCR sequencing (scRNA/BCR-seq) [28–30] and facilitating large-scale clinical studies. To validate this, we curated a diverse external benchmark encompassing healthy, neoplastic, and viral infection states. Under rigorous testing, BCRInsight demonstrated strong resilience to data-source bias and superior cross-scenario generalization, establishing a new standard for BCR sequence modeling.

Furthermore, in paratope prediction tasks, BCRInsight’s multi-head attention mechanism autonomously identifies key antigen-contact residues without requiring explicit three-dimensional (3D) structure supervision. This emergent structural interpretability highlights the capacity of our self-supervised learning to capture biologically meaningful features purely from sequence information. In summary, BCRInsight serves as a powerful, interpretable, and scalable computational engine for antibody sequence analysis, opening new avenues for high-throughput immune repertoire mining and rational antibody design.

## Materials and methods

### Dataset preparation

We curated a comprehensive BCR dataset by integrating and harmonizing raw heavy- and light-chain sequences from multiple public repositories, primarily the Observed Antibody Space (OAS) database [31] and the GSE123158 dataset from GEO [32].

To reduce data redundancy and alleviate distributional bias caused by highly homologous sequences, we performed clustering and deduplication using CD-HIT [33] with a sequence identity threshold of 95%. Crucially, we retained only a single representative sequence from each resulting cluster, completely discarding all other redundant sequences. Because this strict deduplication step was performed prior to splitting the dataset into training, validation, and test sets, it inherently guarantees that the sequence identity between any sequence in the test set and the training set is strictly <95%. Subsequently, a rigorous quality control pipeline was applied. First, sequences lacking critical features, specifically those missing CDR3 regions or incomplete V(D)J gene annotations, were discarded. Second, heavy-chain CDR3 (HCDR3) sequences were filtered to retain only those between 6 and 29 amino acids in length, ensuring exclusion of aberrant V(D)J rearrangements, sequencing artifacts, and low-quality entries. Finally, annotations for germline V(D)J genes and constant regions (C regions) were standardized to ensure consistency across sources.

The resulting high-quality, non-redundant dataset comprised ~89 million unique BCR sequences (67 million heavy chains and 22 million light chains). Detailed multidimensional analysis and statistical overview of the BCR repertoire is provided in Fig. S1, Fig. S8, Table S1, and Table S2. The data were partitioned into training, validation, and test sets using a 90:5:5 ratio. It should be noted that our model is trained exclusively on individual, unpaired single-chain BCR sequences. During model training, heavy and light chains were processed within a unified framework to enable the model to learn shared sequence patterns and immunological features across chain types.

### Overview of BCRInsight

**Figure 1:**
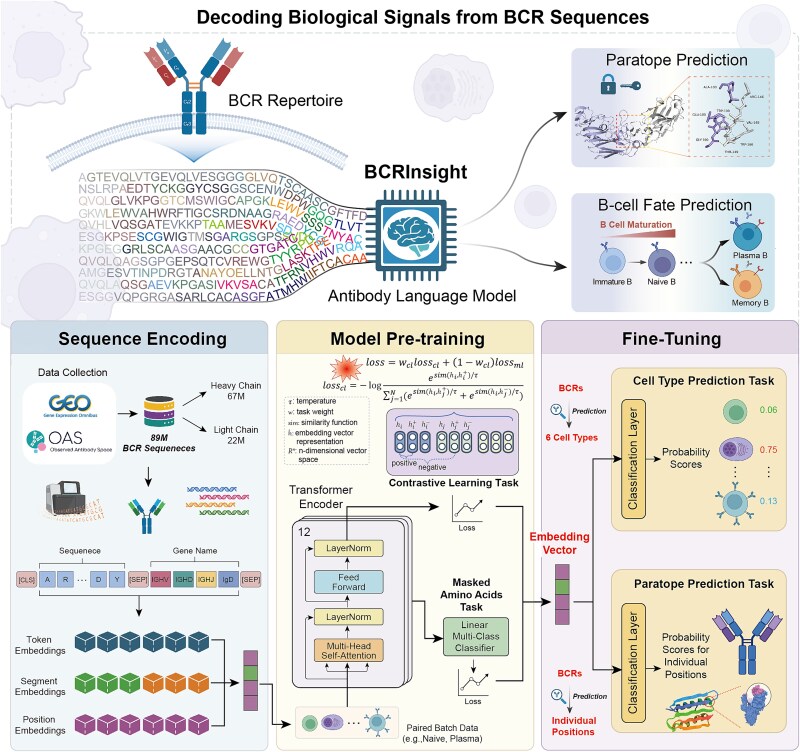
Overview of BCRInsight. “Created with BioRender.com.”

BCRInsight is an antibody-specific PLM implemented based on BERT architecture [22]. To capture the comprehensive biological semantics encoded in BCRs, we designed a composite input representation that integrates both sequence and metadata features. Unlike traditional models that rely solely on amino acid sequences, BCRInsight jointly encodes each BCR sequence together with its corresponding germline gene annotations (V(D)J genes) and isotype information, enabling context-aware representation learning (Fig. 1).

Inspired by “sentence-pair” tasks in natural language processing (NLP), we treat the amino acid sequence and metadata as two semantically related text segments concatenated using a special [SEP] token. An amino acid-level tokenization strategy was adopted to maintain comparability with existing protein language models, such as AntiBERTa [21] and ProtBERT [19], while providing fine-grained residue-level features support for downstream tasks.

For the phenotype-aware CL component, BCR sequences originating from the same B-cell subtypes were defined as positive pairs, while sequences from different subtypes served as negative pairs. This biologically guided training strategy enables the model to capture sequence signatures associated with B-cell developmental stages, functional states, and antigen exposure history, independent of clonal lineage or antigen specificity. Furthermore, given that naive B cells typically predominate in natural immune repertoires [34], we implemented a class-balanced sampling procedure to alleviate subtype imbalance. By assigning relatively equal training weights to high-affinity, functionally mature BCRs and naive counterparts, BCRInsight learns embeddings that encompass both biological diversity and discriminative precision across B-cell phenotypes.

### Model architecture, tokenization, and pretraining

BCRInsight is built on a 12-layer, 12-head Transformer encoder, configured with a hidden dimension of 768 and a feed-forward network dimension of 3072, totaling ~86 million trainable parameters. A 260-token vocabulary was defined that merges 20 amino acids, 230 V(D)J gene symbols, five isotype labels and five special tokens ([CLS], [PAD], [MASK], [SEP], [UNK]). Importantly, our tokenizer design is chain-agnostic; it does not utilize separate vocabularies or specific special tokens to explicitly distinguish between heavy and light chains. Heavy or light-chain sequences were concatenated with their germline and isotype metadata, separated by [SEP], and truncated at 148 tokens; the [CLS] token supplies the sequence-level representation.

The model was pretrained on 80 million human BCR sequences, applying a standard MLM objective: 15% of tokens were chosen at random, of which 80% were replaced with the [MASK] token, 10% with a random amino acid and 10% remained unchanged. This mechanism drives the model to reconstruct the masked positions by relying on bidirectional contextual information, thereby fostering a robust understanding of local and global sequence patterns. For a batch $B$ of sequences $S=\left({s}_1,{s}_2,\dots, {s}_n\right)$, the MLM loss is calculated as:


(1) \begin{equation*} {\mathcal{L}}_{\!\mathcal{mlm}}=-\frac{1}{\left|B\right|}{\sum_{S\in B}}\,\,{\sum_{i\in M}}\mathit{log}\,P\left({s}_i|{S}_{\setminus M}\right) \end{equation*}

Pretraining was conducted for 225 000 steps with a global batch size of 512 on NVIDIA A40 GPUs. We utilized a learning rate schedule with a linear warmup over the first 5% steps to a peak of 1 × 10^−4^, followed by a plateau-based decay strategy (decayed by 0.3 after three steps without validation loss improvement) (Fig. S2). Other detailed configurations can be found in Table S4.

### Contrastive learning for B-cell receptor sequence representation

To capture the functional semantics embedded within BCR sequences, we supplemented MLM with supervised CL driven by B-cell subtype labels. BCRs of the same subtype (naive, memory, plasma, etc.) were treated as positive pairs; sequences from different subtypes served as negatives. This design enables the model to amplify feature discrepancies between developmental stages and constructs a highly discriminative, interpretable latent space.

During training, we employed mini-batch sampling where each batch contained sequences from multiple B-cell subtypes. The model encodes these sequences to obtain embedding vectors ${h}_i$ and ${h}_j$. Similarity was measured using cosine similarity:


(2) \begin{equation*} sim\left({h}_i,{h}_j\right)=\frac{h_i\cdotp{h}_j}{\left\Vert{h}_i\right\Vert \left\Vert{h}_j\right\Vert } \end{equation*}

The contrastive loss aims to maximize the similarity between positive pairs relative to negative pairs, formulated as:


(3) \begin{equation*} {\mathcal{L}}_{\!\mathcal{cl}}=-\mathit{log}\frac{e^{\mathrm{sim}\left({h}_i,{h}_i^{+}\right)/\tau }}{\sum_{j=1}^N{e}^{\mathrm{sim}\left({h}_i,{h}_j^{+}\right)/\tau }+{e}^{\mathrm{sim}\left({h}_i,{h}_j^{-}\right)/\tau }} \end{equation*}

where ${h}_i^{+}$ denotes the positive pair for anchor $i$, and $N$ represents the number of samples in the batch. This enforces intra-subtype compactness and inter-subtype separation, aligning the latent space with B-cell maturation trajectories without requiring structural or clonal information.

### Total loss function

The overall training objective is a weighted sum of the MLM and CL losses, designed to balance contextual reconstruction with semantic discrimination:


(4) \begin{equation*} \mathcal{L}=\left(1-{w}_{cl}\right){\mathcal{L}}_{\!\mathcal{mlm}}+{w}_{cl}{\mathcal{L}}_{\!\mathcal{cl}} \end{equation*}

where ${w}_{cl}$ is a hyperparameter controlling the contribution of the contrastive loss. This dual-objective framework significantly enhances BCRInsight’s ability to encode B-cell functional semantics, providing a powerful representational tool for downstream tasks such as subtype identification and paratope inference.

## Results

### BCRInsight captures biologically meaningful representations

To evaluate the capability of BCRInsight in encoding biologically relevant features, we established a rigorous evaluation framework with strict partitioning between training and test datasets to ensure robust generalizability. To decouple the contributions of different sequence features and identify the optimal balance between computational efficiency and representational power, two distinct input strategies were compared during pre-training: (i) a full-length unpaired strategy, which jointly encodes complete, unpaired BCR sequences with V(D)J gene and isotype tokens to preserve the global structural context and germline framework; and (ii) an HCDR3-focused strategy, which integrates the HCDR3 sequence with the same V(D)J and isotype metadata, emphasizing the high-frequency variation patterns within the core antigen-binding domain while strictly maintaining crucial developmental context.

**Figure 2:**
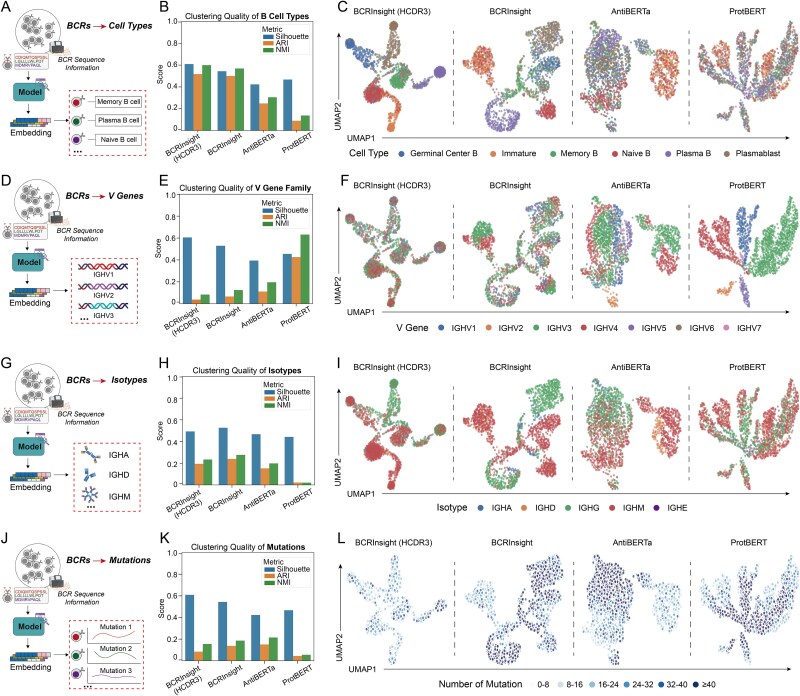
UMAP visualization and clustering evaluation of BCR embedding vectors generated by four models. (A) Schematic diagram for cell type prediction; (B) quantification metrics results of cell type prediction; (C) UMAP projections colored by B-cell type; (D) schematic diagram for V gene prediction; (E) quantification metrics results of V gene prediction; (F) UMAP projections colored by V gene family; (G) schematic diagram for isotype prediction; (H) quantification metrics results of isotype prediction; (I) UMAP projections colored by isotype; (J) schematic diagram for mutation prediction; (K) quantification metrics results of mutation prediction; (L) UMAP projections colored by the number of mutations. “Created with BioRender.com.”

Following pretraining, we extracted the global representations (via the [CLS] token) from a held-out test set of 2000 sequences and visualized them using UMAP (Fig. 2, Fig. S4). Despite the additional contextual information contained in the full-length strategy, the CDR3-specific strategy achieved comparable separation of biologically meaningful groups. This suggests that key functional semantics of B-cell development and differentiation are predominantly encoded within the CDR3 region.

We next benchmarked BCRInsight against state-of-the-art (SOTA) protein language models, AntiBERTa [21] and ProtBERT [19], to quantify embedding performance using clustering metrics (Adjusted Rand Index, ARI; Normalized Mutual Information, NMI; Silhouette Score; see Supplementary Note). BCRInsight markedly outperformed both AntiBERTa and ProtBERT in distinguishing B-cell subtypes and isotypes. As illustrated in the UMAP projections (Fig. 2C and I), while AntiBERTa and ProtBERT produced overlapping embeddings, BCRInsight generated well-segregated clusters corresponding to major B-cell subtypes (naive, memory, plasma) and isotypes (IgM, IgG, IgA). Quantitative metrics (Fig. 2B and H) confirmed these observations, indicating that the phenotype-aware CL framework effectively captures semantic signals highly correlated with immune functional states.

Interestingly, in V-gene family clustering (Fig. 2D–F), BCRInsight exhibited slightly lower performance compared to ProtBERT and AntiBERTa. This reflects a deliberate design trade-off: while general-purpose PLMs emphasize low-level sequence homology, BCRInsight prioritizes high-level functional semantics. Because BCRs originating from distinct V-gene families may converge to similar functional states during affinity maturation [35], the observed reduction in germline-based separation suggests that BCRInsight captures functional semantics beyond mere genetic similarity. Additionally, BCRInsight accurately captured continuous gradients in SHM counts (Fig. 2J–L), illustrating its sensitivity to subtle mutational patterns across immune repertoires. Finally, token-level analysis (Fig. S1, Fig. S3) revealed clear differentiation between amino acids, gene segments, and isotypes in the embedding space, highlighting BCRInsight’s fine-grained interpretability and confirming its capacity to learn a biologically meaningful “vocabulary” of antibody sequences.

### Self-attention mechanism implicitly captures 3D structural constraints

The core advantage of the Transformer architecture [14] lies in its multi-head self-attention mechanism, which captures long-range dependencies within sequences, thereby establishing cross-residue structural and functional associations. Comprising 12 layers with 12 attention heads per layer, BCRInsight is designed to process antibody sequence features concurrently across varying levels of abstraction [36].

**Figure 3:**
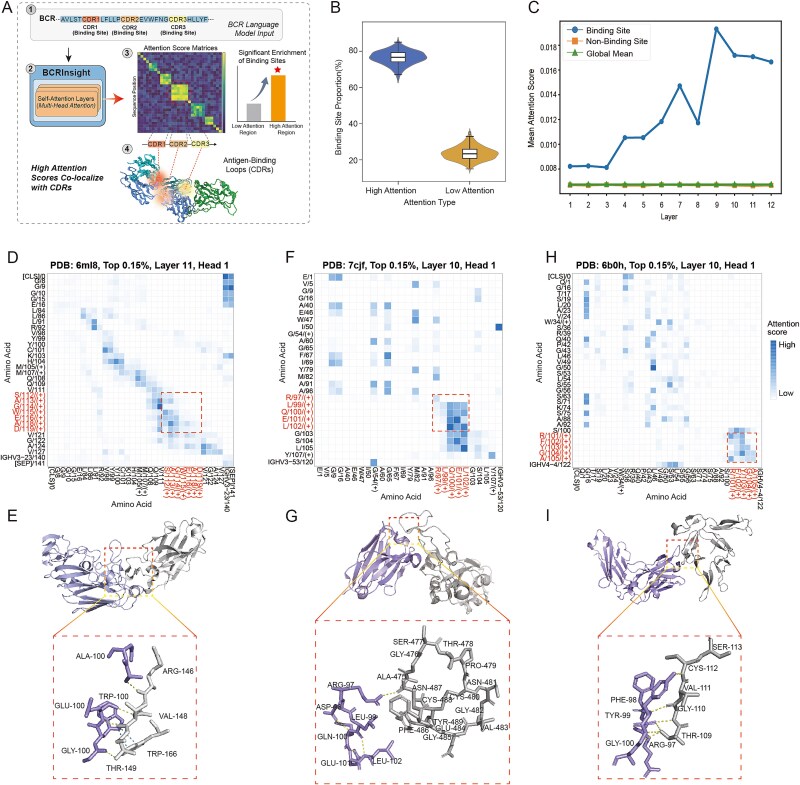
Self-attention mechanism reveals underlying antibody structural contacts and antigen-binding interfaces. (A) Schematic diagram for explainable analysis using BCRInsight; (B) comparison of paratope enrichment between high- and low-attention regions; (C) trajectory of mean attention strength for antigen-binding residues and non-binding residues across model layers 1–12; (D–I) validation of the correspondence between self-attention heatmaps and antibody–antigen complex crystal structures. (D, E) broadly neutralizing influenza antibody C05 in complex with H1N1 hemagglutinin (PDB: 6ML8); (F, G) SARS-CoV-2 neutralizing antibody S309 in complex with the RBD (PDB: 7CJF); (H, I) malaria transmission-blocking antibody 1262 in complex with the Pfs25 protein (PDB: 6B0H). “Created with BioRender.com.”

To investigate the intrinsic relationship between attention weights and 3D spatial structures, we curated a high-quality validation dataset from the SabDab database [37], consisting of 900 antibody sequences with explicitly annotated paratopes. Based on this dataset, we systematically quantified the attention distribution of BCRInsight across residues (Fig. 3A). Statistical analysis revealed a significant positive correlation between attention weights and functional sites: among the top 1% of attention scores, 77% of residues corresponded to true antigen-binding positions, compared with ~23% in low-attention regions (Fig. 3B). These patterns indicate that BCRInsight’s attention mechanism possesses inherent biological selectivity, preferentially focusing on functional regions critical for antigen recognition.

Further layer-wise analysis illuminated the hierarchical evolution of representational learning process. The average attention intensity of antigen-binding residues increased progressively with network depth, peaking at Layer 9, whereas non-binding residues remained consistently low attention across all layers (Fig. 3C). This “hierarchical progression” implies that deeper network layers transition from capturing low-level sequence syntax to encoding high-level structural semantics, autonomously learning spatial conformation underpinning antigen recognition.

To provide visual evidence for these structural correlations, we projected attention heatmaps onto antibody–antigen complex crystal structures. For the broadly neutralizing influenza antibody C05 (PDB: 6ML8 [38]) (Fig. 3D and E), high-weight residue pairs identified by deep attention heads corresponded precisely to long-range contacts at the hemagglutinin interface. Comparable patterns were observed for the SARS-CoV-2 neutralizing antibody S309 (PDB: 7CJF [39]) (Fig. 3F and G) and the malaria-blocking antibody 1262 (PDB: 6B0H [40]) (Fig. 3H and I), where high-attention hotspots were enriched within core paratope regions and structural support sites. The complete attention heatmap and other detialed information are shown in Fig. S5, Fig. S6, Fig. S7. These findings align with observations from related antibody PLMs such as Sapiens and AntiBERTa, confirming that large-scale PLMs can recover 3D structural principles from 1D sequence data even in the absence of explicit structural supervision.

In summary, the self-attention mechanism of BCRInsight not only captures long-range constraints reflecting spatial folding but also precisely localizes functional residues. These results clarify the interpretability basis of deep antibody language models and provide a novel computational framework for inferring structural and functional properties of antibodies directly from sequence information.

### Achieving state-of-the-art performance in paratope prediction tasks

To rigorously evaluate BCRInsight’s capacity for paratope prediction, we curated a benchmark dataset derived from the SabDab database [37]. Following a multi-stage filtering pipeline, including redundancy removal, sequence consistency verification, and precise chain annotation, we obtained 900 high-confidence antibody sequences with explicit paratope labels, establishing a reliable foundation for performance evaluation under realistic biological scenarios. We benchmarked BCRInsight against nine SOTA methods: ProABC-2 [41], Parapred [42], ProtBERT [19], Sapiens [23], AntiBERTa [21], AntiBERTa2 [43], AbLang [44], AbLang2 [45], and BALM [46].

**Figure 4:**
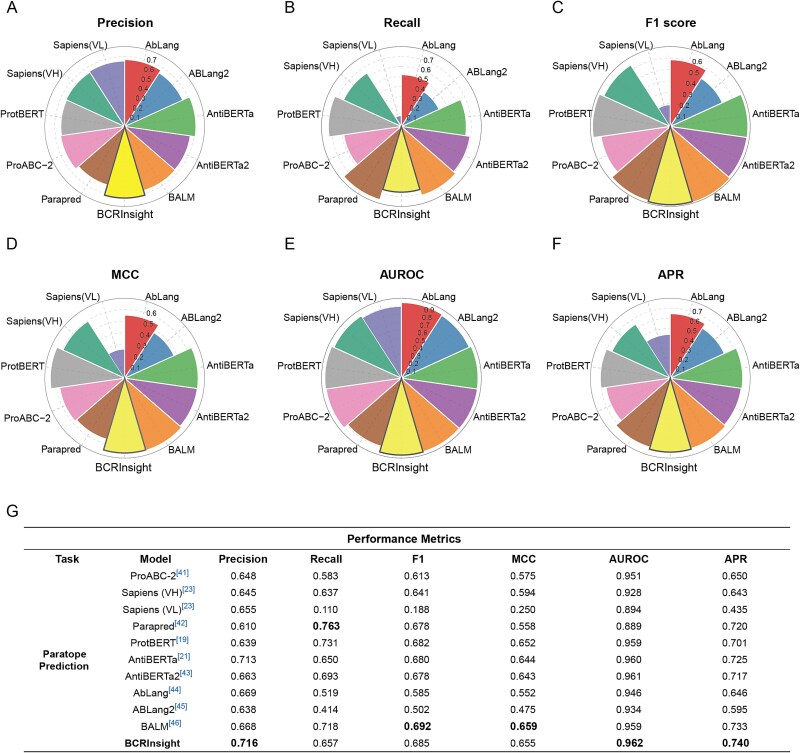
Benchmarking paratope prediction performance. Radar chart comparison of BCRInsight against nine baseline models (ProABC-2, Parapred, ProtBERT, Sapiens, AntiBERTa, AntiBERTa2, AbLang, AbLang2 and BALM) across six key evaluation metrics: Precision, Recall, F1, MCC, AUROC, and APR.

Across six standard evaluation metrics, BCRInsight consistently achieved superior or near-optimal performance (Fig. 4). In the radar chart (Fig. 4), BCRInsight encompasses the largest area, reflecting its comprehensive advantage across metrics. Specifically, Parapred achieved the highest Recall (0.763), while BALM marginally led in the F1 score (0.692) and MCC (0.659). Despite a slight disadvantage in these specific areas, BCRInsight delivered highly comparable performance with an F1 score of 0.685 and an MCC of 0.655. Given the inherent class imbalance in paratope prediction, where true antigen-binding residues constitute only a small fraction of sequence positions, BCRInsight’s remarkable Area Under the ROC Curve (AUROC: 0.962) and strong Matthews Correlation Coefficient (MCC: 0.655, highly competitive with BALM’s 0.659) highlight its capacity to robustly identify rare functional sites amidst a vast background of non-binding residues. Furthermore, its high Average Precision (APR: 0.740) demonstrates the model’s strong capability to rank true binding residues with high confidence.

This outstanding performance arises from the synergy between BCRInsight’s attention-based architecture and balanced data strategy. The model exhibits a hierarchical attention focusing behavior, where deeper layers progressively concentrate attention on paratope regions, effectively reconstructing the antigen contact interface without explicit structural supervision. Simultaneously, the class-balanced sampling strategy applied during pre-training proved critical. By mitigating the natural repertoire bias dominated by naive B cells, this strategy prevents overfitting to germline features and promotes the learning of high-frequency binding motifs generated through antigen-driven selection and affinity maturation. Crucially, while BALM demonstrates comparable efficacy in the specific task of paratope prediction, BCRInsight distinguishes itself through superior computational efficiency and broader functional versatility. As a significantly more lightweight architecture, BCRInsight requires fewer computational resources, yet delivers commensurate or even enhanced performance across key metrics. Furthermore, beyond paratope prediction, BCRInsight is systematically engineered to support a much richer diversity of downstream tasks. In summary, BCRInsight demonstrates new SOTA performance and interpretability in paratope prediction, combining deep structural awareness with biological robustness across B-cell developmental stages. These results establish BCRInsight as a powerful computational engine for rational antibody design and functional repertoire analysis.

### Assessing generalization across clinically divergent immune landscapes

Bulk BCR-seq remains the workhorse for profiling antibody repertoires, but the absence of cellular metadata prevents direct assignment of B-cell subpopulations or functional states. Existing computational methods therefore resort to IGHV SHM thresholds that segregate naive (low-mutated, lgM/D) from antigen-experienced B cells, yet fail to resolve memory B cells versus antibody-secreting cells (ASCs) [47]. Recently emerged machine-learning models such as BCR-SORT [48] exploit HCDR3 patterns; however, they are restricted to three coarse lineages (naive, memory, ASCs) and the CNN/LSTM-based architectures capture only local motifs [4]. scRNA/BCR-seq can directly link the transcriptome of individual B cells to their cognate receptor sequences, but its high cost and poor BCR recovery (one to two orders of magnitude fewer unique clones than bulk BCR-seq) preclude large-scale and comprehensive repertoire investigation [6, 49, 50].

**Figure 5:**
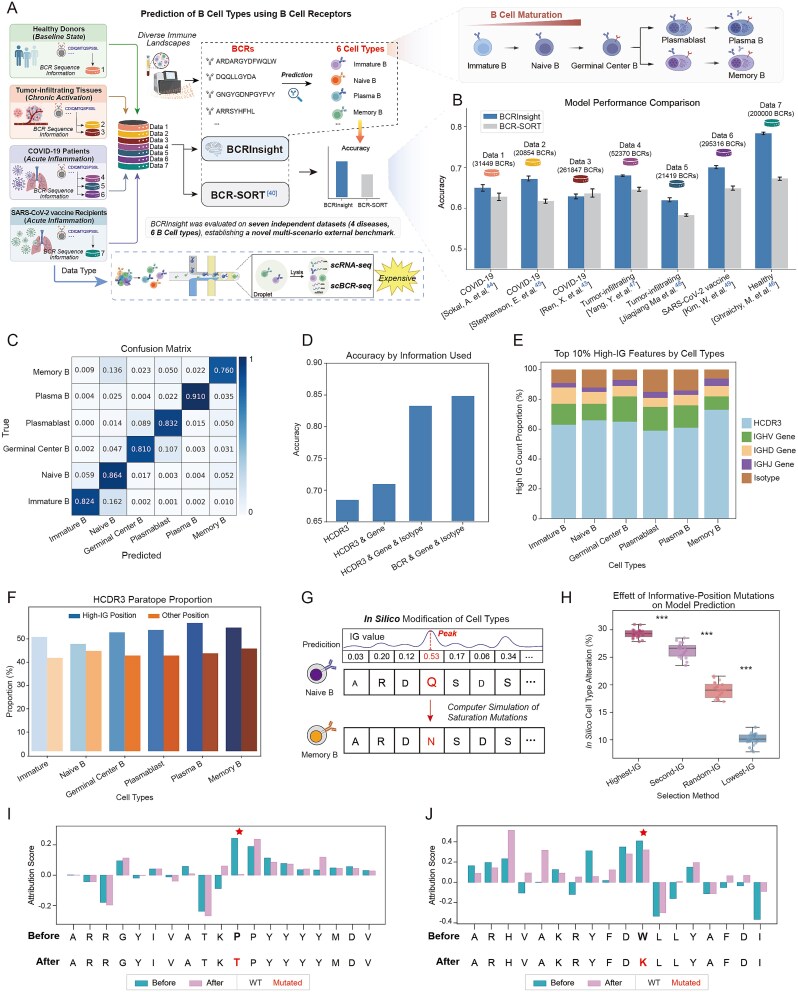
B-cell subpopulation prediction and model interpretability analysis using BCRInsight. (A) Schematic illustration of the workflow associating B-cell subpopulations with BCR sequences using BCRInsight; (B) comparison results of BCRInsight and BCR-SORT on the new benchmark covering healthy individuals, tumor-infiltrated tissues and COVID-19 patients; (C) confusion matrix on the internal test set; (D) ablation study results of the impact of different model inputs on model accuracy; (E) feature importance analysis based on IG, displaying the distribution of the top 10% contributing features. (F) Enrichment analysis of antigen-binding sites (paratopes) within high-IG regions; (G) *In silico* saturation mutagenesis analysis; (H) impact of key residue mutations with different IG scores on prediction results. Asterisks indicate statistical significance, demonstrating the critical role of high-IG residues in maintaining B-cell subpopulation identity; (I, J) Case examples of IG score variations pre- and post-mutation of high-IG residue. “Created with BioRender.com.”

To quantify whether BCRInsight can deliver single-cell level resolution from bulk BCR repertoires, we assembled an external validation set of ~0.5 million HCDR3 sequences curated from matched scRNA/BCR-seq data [51–57]: (i) healthy PBMCs (baseline), (ii) tumor-infiltrating B cells (chronic activation), and (iii) serial samples from COVID-19 and early SARS-CoV-2 patients (acute inflammation) (Fig. 5A). This resource provides the clinically diverse benchmark currently available for six canonical B-cell subtype prediction tasks.

When benchmarked against BCR-SORT [48], currently the most representative sequence-based B-cell subtype prediction model (Fig. 5A), BCRInsight (HCDR3) reached a macro-averaged accuracy of 0.688, outperforming the 0.664 accuracy of BCR-SORT (Table 1, Fig. 5B). The confusion matrix constructed on the pre-reserved held-out test set exhibited class-wise recalls of 0.76–0.91, with the most frequent off-diagonal errors <5% (Fig. 5C), confirming robust generalization across clinically divergent immune landscapes. Furthermore, the ablation study conducted on this same pre-reserved dataset revealed that HCDR3 alone retains ≥98% of the accuracy obtained with full-length heavy/light sequences while eliminating the complexity of full-length inputs, thus achieving an optimal accuracy-efficiency trade-off (Table S3, Fig. 5D).

**Table 1:** B-cell type prediction results of BCRInsight.

Dataset	Model	Precision	Recall	F1	MCC
PMID:33657410 [51] COVID-19	BCR-SORT [48]	0.676	0.638	0.641	0.580
	**BCRInsight**	0.655	0.628	0.621	0.551
PMID:33571429 [52] COVID-19	BCR-SORT	0.701	0.627	0.632	0.566
	**BCRInsight**	0.702	0.653	0.661	0.594
PMID:33879890 [53] COVID-19	BCR-SORT	0.636	0.603	0.607	0.543
	**BCRInsight**	0.683	0.669	0.666	0.608
PMID:34661527 [54] Healthy	BCR-SORT	0.691	0.668	0.668	0.608
	**BCRInsight**	0.783	0.778	0.774	0.745
PMID:39047727 [55] Tumor-infiltrating	BCR-SORT	0.668	0.644	0.645	0.584
	**BCRInsight**	0.686	0.681	0.678	0.622
PMID:38696569 [56] Tumor-infiltrating	BCR-SORT	0.611	0.574	0.581	0.512
	**BCRInsight**	0.618	0.589	0.595	0.534
PMID:35168246 [57] SARS-CoV-2 vaccine	BCR-SORT	0.668	0.641	0.638	0.579
	**BCRInsight**	0.691	0.692	0.689	0.639

These results demonstrate that large-scale, phenotype-aware pretraining of BCRInsight extracts conserved functional signatures that remain stable regardless of disease context and data source biases, establishing a sequence-only classifier whose reliability approaches the level required for routine clinical specimen deconvolution.

### Deciphering feature contributions and causal residues via integrated gradients

To quantify the contribution of each input token to model predictions, we employed Integrated Gradients (IG) [58, 59] (Supplementary Note 1.2) with a zero-embedding baseline and 200 interpolation steps, implemented in Captum [60]. IG scores were summed per residue and ranked; the top decile was retained for analysis.

Across B-cell subtypes, the HCDR3 region consistently carried the highest aggregate IG attribution (Fig. 5E). Naive and plasma B cells also exhibited strong isotype signals, reflecting surface IgM/IgD and IgA/IgG dominance respectively [61], whereas memory B cells displayed 20% higher HCDR3 IG score compared to less antigen-experienced naive or immature B cells. This discrepancy suggests that BCRInsight leverages antigen-driven selection signatures embedded in the antigen-binding HCDR3 sequence.

B-cell activation and maturation are accompanied by increased BCR-antigen binding affinity and the accumulation of SHM [62]. Consistent with this mechanism, we observed that high IG values were predominantly assigned to HCDR3 residues associated with antigen binding and SHM. Utilizing Parapred [42] to identify potential paratope residues within HCDR3, we found that the proportion of paratopes was significantly higher among high-IG positions relative to the remaining residues (Fig. 5F). Moreover, as B cells develop from immature through naive to mature states (memory or plasma), the overlap between high-IG positions and Parapred-predicted paratopes progressively increases, mirroring the trajectory of affinity maturation. Thus, without structural supervision, BCRInsight learns to up-weight exactly those residues that confer antigen-binding capacity, validating that the model exploits activation- and maturation-imprinted signatures to discriminate B-cell subtypes.

To determine whether residues with the highest-IG scores are causally required for B-cell subtype prediction, we performed *in silico* saturation mutagenesis [63]. Each of the top IG positions and an equal number of randomly selected positions were individually mutated to all 19 alternative amino acids, and the resulting mutant sequences were re-submitted to the trained model (Fig. 5G). Substitutions at the highest-IG positions decreased the original subtype probability 3.4-fold more frequently than random-residue substitutions (Fig. 5H), demonstrating that these highest-IG residues are functional determinants of B-cell identity rather than statistical artifacts. To further visualize the functional impact of these computational mutations, we randomly selected two examples for *in silico* perturbation validation of the high-IG positions. It should be noted that this validation is a purely computational analysis designed to probe the model’s learned representations, without accompanying wet-lab experiments. The computational evaluation (Fig. 5I and J) showed that when the high-IG positions were mutated, the IG scores of other positions in the sequence all exhibited significant alterations. This demonstrates that high-IG positions alter the overall feature correlation of the sequence, thereby indirectly affecting the contribution of other positions to B-cell subtype prediction.

## Discussion

Natural language encapsulates complex syntactic structures and semantic logic through text. Similarly, BCR sequences are not random arrangements of amino acids but constitute a “biological language” governed by rules that orchestrate folding, stability, and antigen specificity. In this study, we conceptualized BCR sequences through the lens of NLP: the combinatorial arrangement of amino acids constitutes the syntax, determining physicochemical constraints; while specific patterns shaped by antigen-driven selection constitute the semantics, defining immune function and cellular phenotypes. Under this paradigm, BCRInsight functions not merely as a sequence model, but as a decoder capable of self-supervised learning to unravel the intrinsic evolutionary rules of antibodies from massive datasets.

Our findings demonstrate that the embeddings generated by BCRInsight successfully encode multidimensional biological features, including mutation load, cell subtypes, isotypes, and V-gene families. This highlights the unique advantage of combining the Transformer architecture with phenotype-aware CL. Unlike traditional methods reliant on hand-crafted features (e.g. k-mers or physicochemical properties), BCRInsight automatically captures high-order non-linear features—often elusive to manual definition—by explicitly clustering sequences with similar phenotypes during pre-training. This end-to-end learning paradigm shifts the burden from laborious feature engineering to the pre-training phase, enabling SOTA accuracy in downstream tasks.

A distinct advantage of Transformers over “black-box” models lies in the interpretability provided by the attention mechanism. Although no 3D structural supervision was introduced during training, we observed a high overlap between high-attention regions and ground-truth antigen-binding sites. This phenomenon suggests an emergence of structural awareness, implying that to maximize the accuracy of masked token reconstruction, the model is driven to implicitly reconstruct residue–residue contact maps. While current attention signals contain noise and cannot fully replace crystallographic determination, they offer valuable intuitive clues for understanding “how sequence determines function”, confirming that deep language models capture biological signals transcending simple sequence motifs.

The most transformative potential of BCRInsight lies in its precision in deconvoluting B-cell subpopulations. Traditionally, resolving the fine-grained composition of immune repertoires has relied on expensive single-cell sequencing or laborious flow cytometry. Our model offers an efficient computational alternative: the ability to infer specific subpopulation proportions solely from cost-effective bulk sequencing data. This breakthrough significantly reduces the financial and temporal costs of large-scale clinical studies and mitigates information loss caused by cell dropout or insufficient sampling in single-cell technologies. Furthermore, by constructing an independent benchmark spanning healthy baselines, chronic cancer inflammation, and acute COVID-19 infection, we demonstrated that BCRInsight captures core functional commonalities of B cells that persist across disease states, marking a pivotal step toward practical clinical translation.

Despite these encouraging results, several limitations warrant acknowledgment. First, BCRInsight currently treats BCRs as independent entities, lacking the repertoire-level context that would account for the presence of clonally related sequences from the same source. Second, the model cannot distinguish between two distinct B-cell subtypes encoded by identical BCR sequences—a scenario that may arise when cellular maturation significantly precedes sequence divergence or due to convergent evolution.

Looking ahead, we aim to address these challenges by integrating paired-chain data and multi-modal information, such as T-cell receptor sequences or gene expression profiles. Future iterations will employ more sophisticated fine-tuning strategies to extend BCRInsight to generative tasks, such as the *de novo* design and optimization of disease-specific antibodies. Ultimately, we envision a transition from merely reading the immune language to actively writing it, paving the way for next-generation immunotherapeutics.

## Conclusion

In summary, this study presents BCRInsight, an antibody-specific protein language model that effectively bridges the gap between BCR sequence data and immune functional phenotypes. By leveraging a novel CL strategy and a massive, curated dataset, BCRInsight achieves superior performance in parsing B-cell subpopulations and identifying antigen-binding sites, while exhibiting robust generalization across diverse clinical landscapes. Beyond its predictive power, the model offers interpretable insights into the structural constraints of antibodies. As a powerful computational engine, BCRInsight provides a scalable, cost-effective solution for immune repertoire analysis, holding significant promise for advancing rational antibody design and precision medicine.

**Key Points**

- We present BCRInsight, a BCR language model integrating phenotype-aware contrastive learning to capture deep functional semantics, achieving state-of-the-art performance in paratope prediction.
- Our approach enables precise deconvolution of B-cell subpopulations from bulk BCR sequencing data, offering a scalable and cost-effective alternative to single-cell sequencing.
- Analysis of the attention mechanism reveals structural interpretability, identifying antigen-binding sites without 3D structural supervision.
- Comprehensive benchmarking across diverse clinical cohorts (healthy, neoplastic, and COVID-19) validates the model’s robust generalization compared to existing methods.

## Supplementary Material

BCRInsight_SM_revised_bbag154

## Data Availability

Source code and data used in this study are available at https://github.com/nullptr-error/BCRInsight-model.
