# Supplementary material for: BCRInsight: an antibody language model to decode biological signals from BCR sequences
Source: Brief Bioinform. 2026 Apr 14;27(2):bbag154. doi: 10.1093/bib/bbag154 (PMC13076941; doi:10.1093/bib/bbag154)
Supplement: BCRInsight_SM_revised_bbag154 [file bcrinsight_sm_revised_bbag154.pdf]

Supplementary Material for "BCRInsight: A Language Model to Decode Biological Signals from BCR Sequences"

Hailong Zhao[1,2]‡; Shang Lou[1,2]‡; Xuhua Li[1,2]‡; Yiyang Gao[3]; Hongcang Gu[1,2*]; Fan Zhang[1,2*]

[1] Anhui Province Key Laboratory of Medical Physics and Technology, Institute of Health and Medical Technology, Hefei Institutes of Physical Science, Chinese Academy of Sciences, Hefei, 230031, China.

[2] University of Science and Technology of China, Hefei, 230026, China.

[3] HIT Center for Life Sciences, School of Life Science and Technology, Harbin Institute of Technology, Harbin 150080, China

‡ Hailong Zhao, Shang Lou and Xuhua Li have equal first authorship.

[*]Corresponding author(s). fzhang@cmpt.ac.cn (Fan Zhang); gu_hongcang@cmpt.ac.cn (Hongcang Gu)

Content

1 Supplementary Note

1.1 Metrics

1.2 Integrated Gradients (IG)

1.3 Clarification of Input Features Across Downstream Tasks

1.4 Methodological Comparison of BCRInsight with Existing Antibody PLMs

1.5 Source and Harmonization of B-Cell Subtype Labels

2. Supplementary Figures

Figure S1. Multidimensional profiling of BCR repertoire characteristics across B-cell developmental stages.

Figure S2. Training convergence of the BCRInsight model.

Figure S3. Visualization of token embeddings in the BCRInsight vocabulary.

Figure S4. Immunogenicity score evaluation results of the three models

Figure S5. Binding Site Attention Strength and Intra-layer Diversity across Layers of BCRInsight.

Figure S6. Attention Heads Sensitivity to Binding Sites.

Figure S7. BCRInsight interpretability via self-attention scores.

Figure S8. BCR physicochemical landscapes across developmental subsets.

3. Supplementary Tables

Table S1. Statistical Overview of Training Dataset

Table S2. Proportion of antibody types in different cell subsets of the training dataset

Table S3. Ablation study on input attributes of B-cell subtype prediction task

Table S4. Configuration of BCRInsight

Table S5 Clarification of model feature architecture and actual inference inputs in downstream tasks

Table S6 Key differences between BCRInsight and AntiBERTa/Sapiens

Table S7 Harmonization mapping rules and established FACS surface markers for B-cell subtypes.

1. Supplementary Note

1.1 Metrics

To quantitatively assess the quality of representations generated by BCRInsight and baseline models, we performed K-means clustering on the embedding vectors and evaluated the results using three standard metrics: Adjusted Rand Index (ARI), Normalized Mutual Information (NMI), and Silhouette Score. The detailed definitions are as follows:

Adjusted Rand Index (ARI). ARI is a corrected version of the Rand Index (RI) that measures the similarity between two data clusterings (ground truth labels versus predicted clusters). ARI adjusts for chance, ensuring that the score for random clustering is close to 0.0, independent of the number of samples and clusters. Given a set of n samples, let U be the set of true classes and V be the set of clusters predicted by K-means. The ARI is defined as:

$$ARI = \frac{\sum_{ij}\binom{n_{ij}}{2} - [\sum_i\binom{a_i}{2}\sum_j\binom{b_j}{2}]/\binom{n}{2}}{\frac{1}{2}[\sum_i\binom{a_i}{2} + \sum_j\binom{b_j}{2}] - [\sum_i\binom{a_i}{2}\sum_j\binom{b_j}{2}]/\binom{n}{2}} \quad (1)$$

Where n_{ij} denotes the number of samples common to class u_i and cluster v_j. **Range:** [-1, 1]. A score of 1.0 indicates perfect agreement between the clustering result and the ground truth; 0 indicates that the clustering is equivalent to random assignment.

Normalized Mutual Information (NMI). NMI is an information-theoretic measure used to quantify the mutual dependence between true labels and predicted clusters, normalized to the range [0, 1]. The calculation formula is:

$$NMI(U,V) = \frac{2 \times I(U;V)}{H(U) + H(V)} \quad (2)$$

Where $H(U)$ and $H(V)$ represent the entropies of the partitions, and $I(U;V)$ denotes the mutual information. **Range:** [0, 1]. A score of 1.0 indicates perfect correlation; 0 indicates no mutual information.

Silhouette Score. Unlike ARI and NMI, the Silhouette Score does not require ground truth labels. It evaluates the internal validity of clustering by comparing the similarity of a sample to its own cluster (cohesion) against its similarity to other clusters (separation). For a single sample i, the Silhouette coefficient is defined as:

$$s(i) = \frac{b(i) - a(i)}{\max\{a(i), b(i)\}} \quad (3)$$

Where $a(i)$ is the mean distance between sample i and all other points in the same cluster, and $b(i)$ is the mean distance between sample i and all points in the nearest neighboring cluster. **Range:** [-1, 1]. A score close to 1 indicates that the sample is far from neighboring clusters (implying dense and well-separated clusters); 0 indicates overlapping clusters; negative values suggest the sample may have been assigned to the wrong cluster.

1.2 Integrated Gradients (IG)

The IG value is calculated as follows:

$$IG(V^{ij}) = \sum_{k=1}^{l} (V_k^{ij} - \tilde{V}_k^{ij}) \times \sum_{p=1}^{m} \frac{\partial F[\tilde{V} + \frac{p}{m} \times (V - \tilde{V})]}{\partial V_k^{ij}} \times \frac{1}{m} \quad (4)$$

where F denotes the model, V the input features, $\tilde{V}$ the baseline, and m the number of interpolation steps.

1.3 Clarification of Input Features Across Downstream Tasks

The foundational architecture of BCRInsight is designed to jointly encode B-cell receptor (BCR) amino acid sequences alongside their corresponding biological metadata, specifically V(D)J gene usage and isotype tokens. This joint encoding strategy is strictly maintained across our model designs. However, during the evaluation of downstream tasks, the specific combinations of input features were adapted based on two factors: (1) the biological and computational requirements of the specific task (i.e., sequence length), and (2) the inherent availability of metadata within the downstream benchmark datasets.

For the UMAP visualization task, we utilized both the BCRInsight(HCDR3) model and the full-length BCRInsight model, incorporating all native metadata features (V(D)J genes and isotype tags) to provide a comprehensive landscape of the sequence space.

For the B-cell subtype prediction classification task, we employed the BCRInsight(HCDR3) model. As detailed in our feature ablation study (Fig. 5D, Table S3), substituting the full-length sequence with the HCDR3 sequence—while strictly retaining the V(D)J and isotype tokens—

yielded the optimal balance between high classification accuracy and computational efficiency.

For the paratope prediction task, comprehensive global structural context is inherently required to accurately identify antigen-binding interfaces; therefore, the full-length BCRInsight model was utilized. It is critical to note that while the core BCRInsight architecture is designed to process isotype tokens, the standard benchmark dataset used for paratope prediction lacks isotype annotations. Consequently, the actual input provided to the model during inference for this specific task was limited to the full-length BCR sequence and V(D)J gene tokens. The omission of the isotype token was strictly due to downstream dataset limitations, rather than a reversion to a "sequence-only" modeling strategy. The continued integration of V(D)J genes underscores the model's reliance on metadata-aware representations.

To provide absolute transparency regarding our evaluation methodology, the original model input space (training features) and the actual features provided during inference for each downstream task are explicitly summarized in Table S5.

1.4 Methodological Comparison of BCRInsight with Existing Antibody PLMs

While existing pre-trained language models (PLMs) such as AntiBERTa and Sapiens have demonstrated strong capabilities in modeling antibody sequence syntax, they primarily operate on raw amino acid sequences using unsupervised Masked Language Modeling (MLM). Consequently, these models often lack explicit integration of the rich biological context inherent to B-cell receptors (BCRs).

To address this limitation, BCRInsight introduces a phenotype-aware pre-training paradigm. The key methodological differences between BCRInsight and existing baselines are summarized in **Table S6**. Specifically, BCRInsight diverges from previous methods in three core aspects:

Multimodal Inputs and Phenotypic Labels: Instead of relying solely on amino acid sequences, BCRInsight employs a joint encoding strategy that integrates V(D)J gene tokens and isotype tokens. Furthermore, it incorporates curated B-cell type labels as anchors for a supervised Contrastive Learning (CL) objective. This enables the model to integrate

evolutionary origins and developmental stages simultaneously, mapping sequences into a biologically meaningful latent space rather than relying purely on sequence edit distances.

Class-Balanced Sampling: Traditional random sampling from natural repertoires inevitably biases models toward abundant, naive (unmutated) B-cell sequences. BCRInsight utilizes a class-balanced sampling strategy based on B-cell types. This mitigates repertoire bias, prevents overfitting to germline features, and explicitly forces the model to learn critical binding motifs generated during antigen-driven affinity maturation.

Comprehensive Evaluation: By combining the MLM objective with contrastive learning, BCRInsight achieves SOTA performance not only in micro-level structural tasks (e.g., paratope prediction) but also in macro-level, systems-immunology tasks (e.g., cell subtype annotation), demonstrating a more comprehensive understanding of the immune repertoire.

1.5 Source and Harmonization of B-Cell Subtype Labels

A critical component of the phenotype-aware supervised contrastive learning objective in BCRInsight is the fidelity of the B-cell subtype labels. To ensure high representation quality and minimize noisy labels, we exclusively utilized datasets where subtype annotations represent physical, experimental ground truth derived from Fluorescence-Activated Cell Sorting (FACS).

The sequence data and metadata comprising our pre-training corpus were systematically retrieved from the Observed Antibody Space (OAS) database, alongside Mitsunaga's study (*Deep Characterization of the Human Antibody Response to Natural Infection Using Longitudinal Immune Repertoire Sequencing*). The inherent validity of these labels relies on the rigorous experimental quality control (QC) and gating pipelines established by the original authors prior to data deposition.

Because different independent studies may employ slightly varying nomenclature for B-cell populations, we performed a comprehensive harmonization process. We manually reviewed the source literature associated with each dataset to extract the exact FACS surface marker gating strategies utilized during cell isolation. Based on these documented physical phenotypic profiles, the original annotations were systematically mapped into six standardized B-cell subtypes: Immature, Naive, Memory, Plasma (ASC), Plasmablast (PB), and Germinal

Center B (GCB) cells.

The explicit mapping rules and the defining FACS surface markers aggregated from the source literature are detailed in Table S7.This mapping strategy ensures that our contrastive loss function operates on biologically robust boundaries grounded in standardized experimental immunology.

2. Supplementary Figures

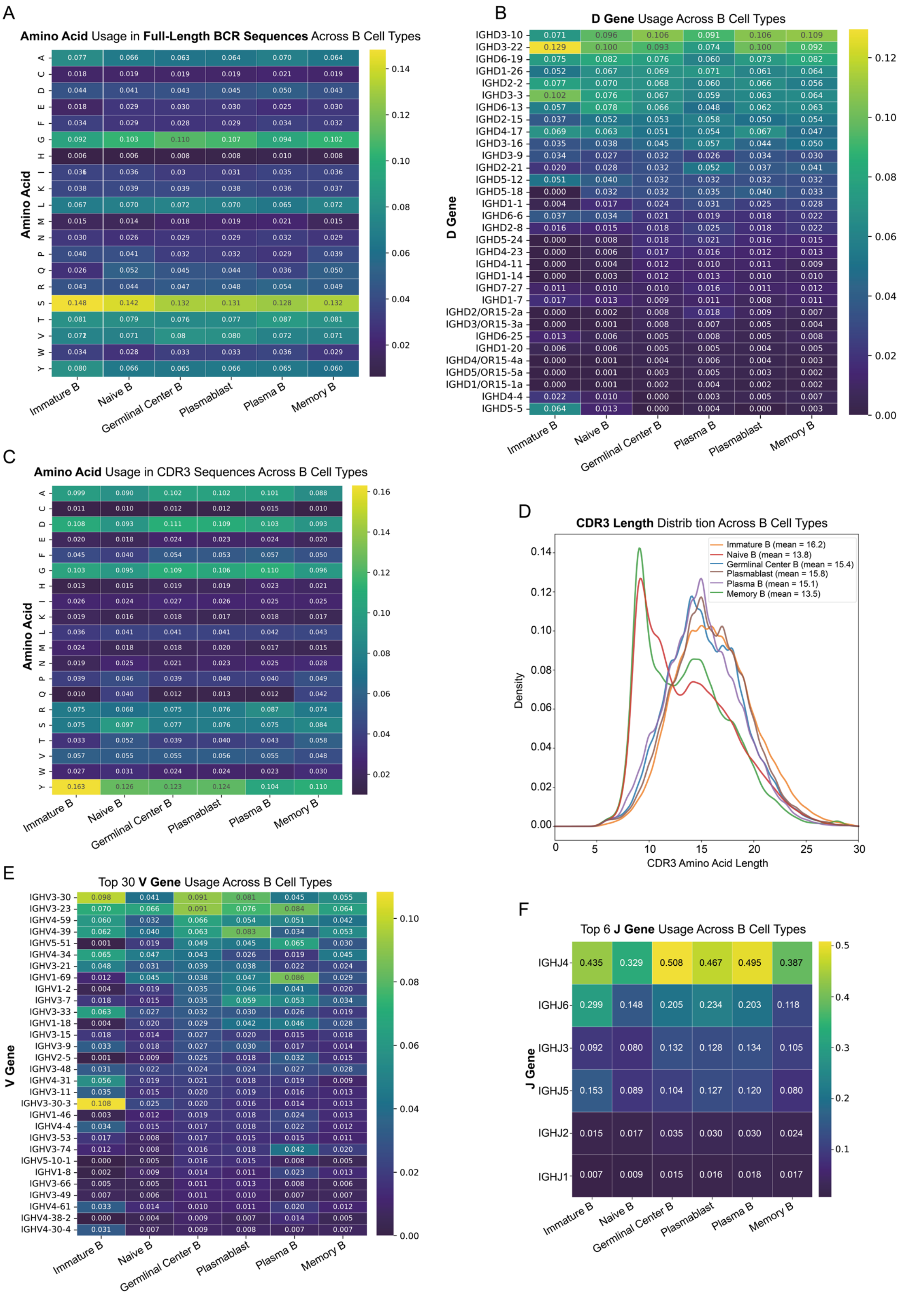

Figure S1. Multidimensional profiling of BCR repertoire characteristics across B-cell developmental stages. (A) Heatmap of amino acid usage in full-length BCR sequences across

six B-cell developmental subsets. (B) Frequency distribution of D gene segment usage across B-cell types. (C) Heatmap showing amino acid usage patterns specifically within CDR3 sequences. (D) Density distribution plots of CDR3 amino acid lengths across developmental stages. (E) Usage frequencies of the top 30 V genes across B-cell subsets. (F) Frequency distribution of the top 6 J genes across stages. All data are categorized by six distinct B-cell types: Immature B, Naive B, Germinal Center B, Plasmablast, Plasma B, and Memory B.

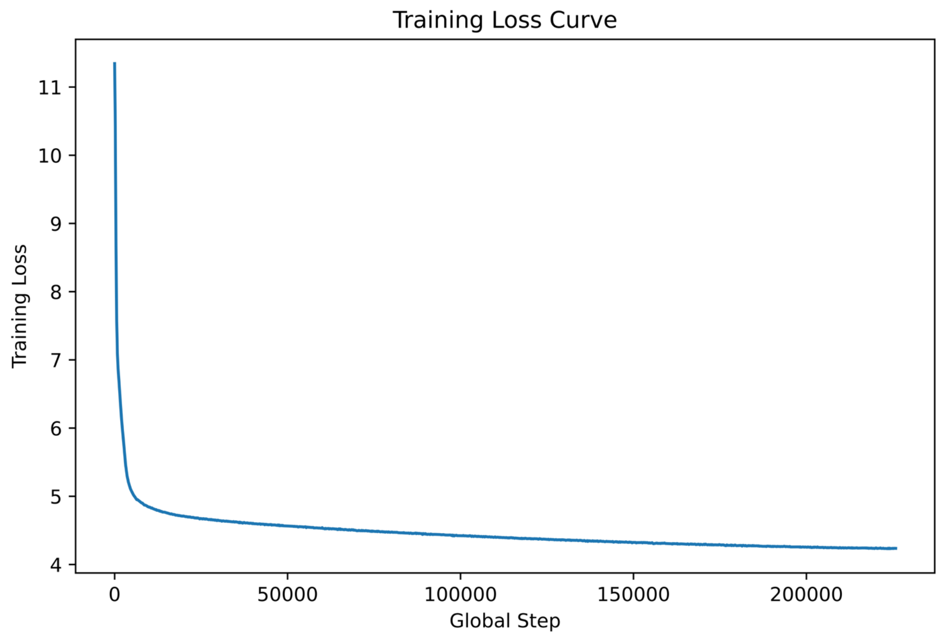

Figure S2. Training convergence of the BCRInsight model.

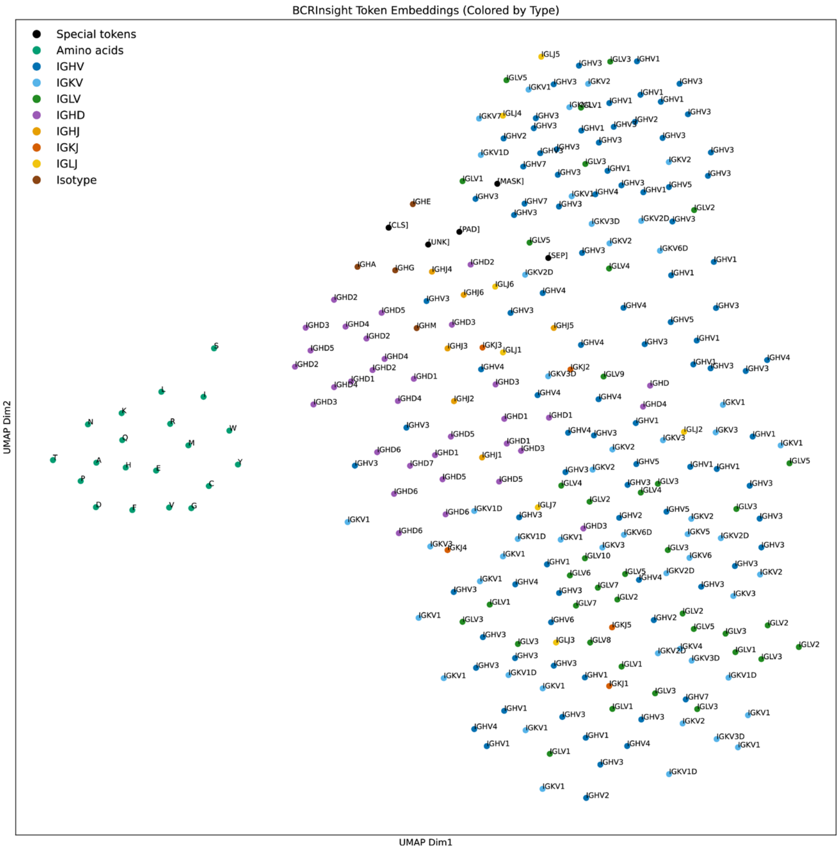

Figure S3. Visualization of token embeddings in the BCRInsight vocabulary.

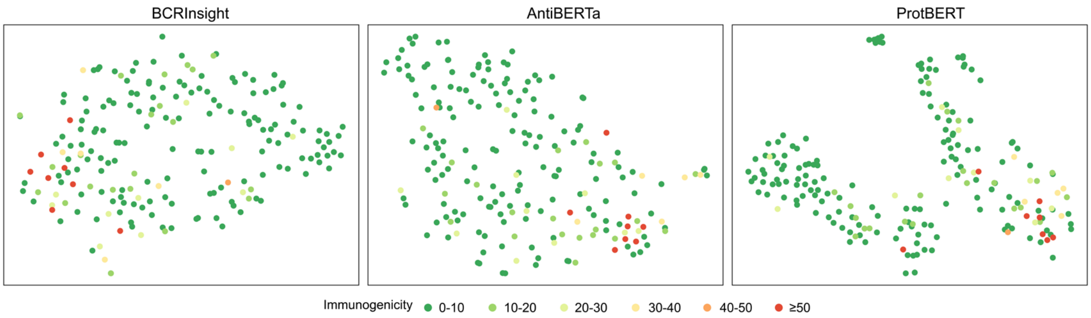

Figure S4. Immunogenicity score evaluation results of the three models.

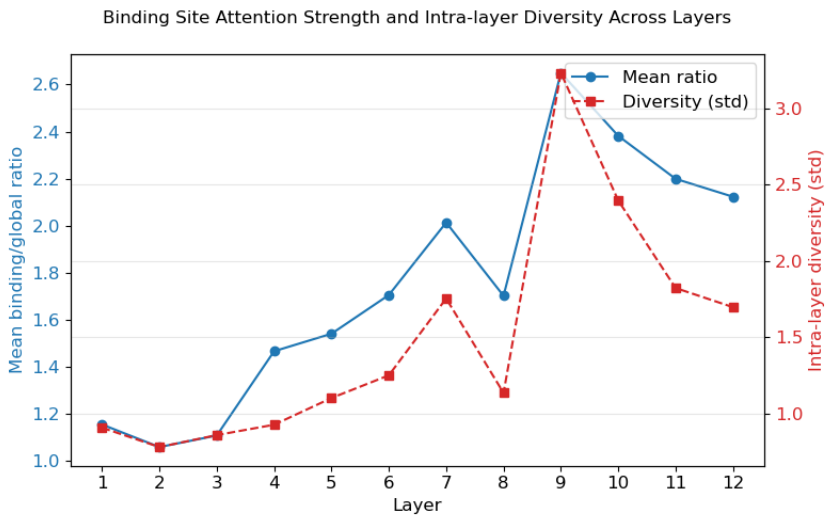

Figure S5. Binding Site Attention Strength and Intra-layer Diversity across Layers of BCRInsight.

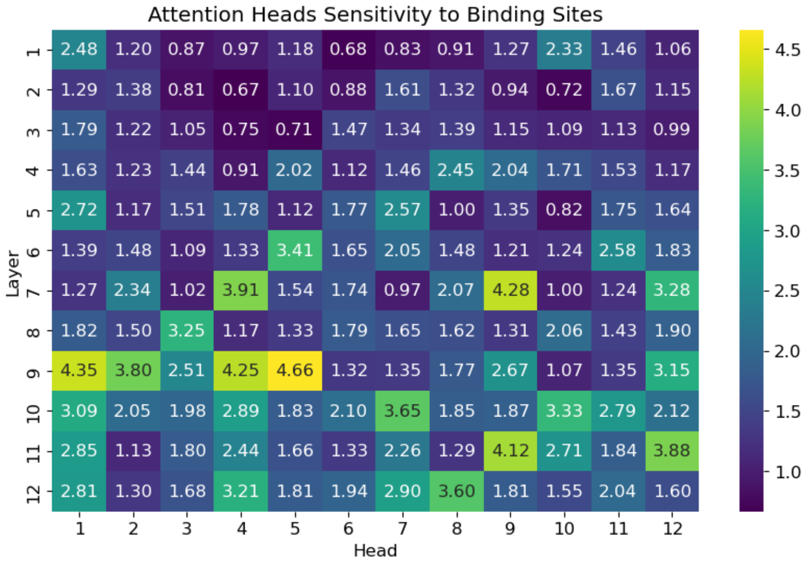

Figure S6. Attention Heads Sensitivity to Binding Sites.

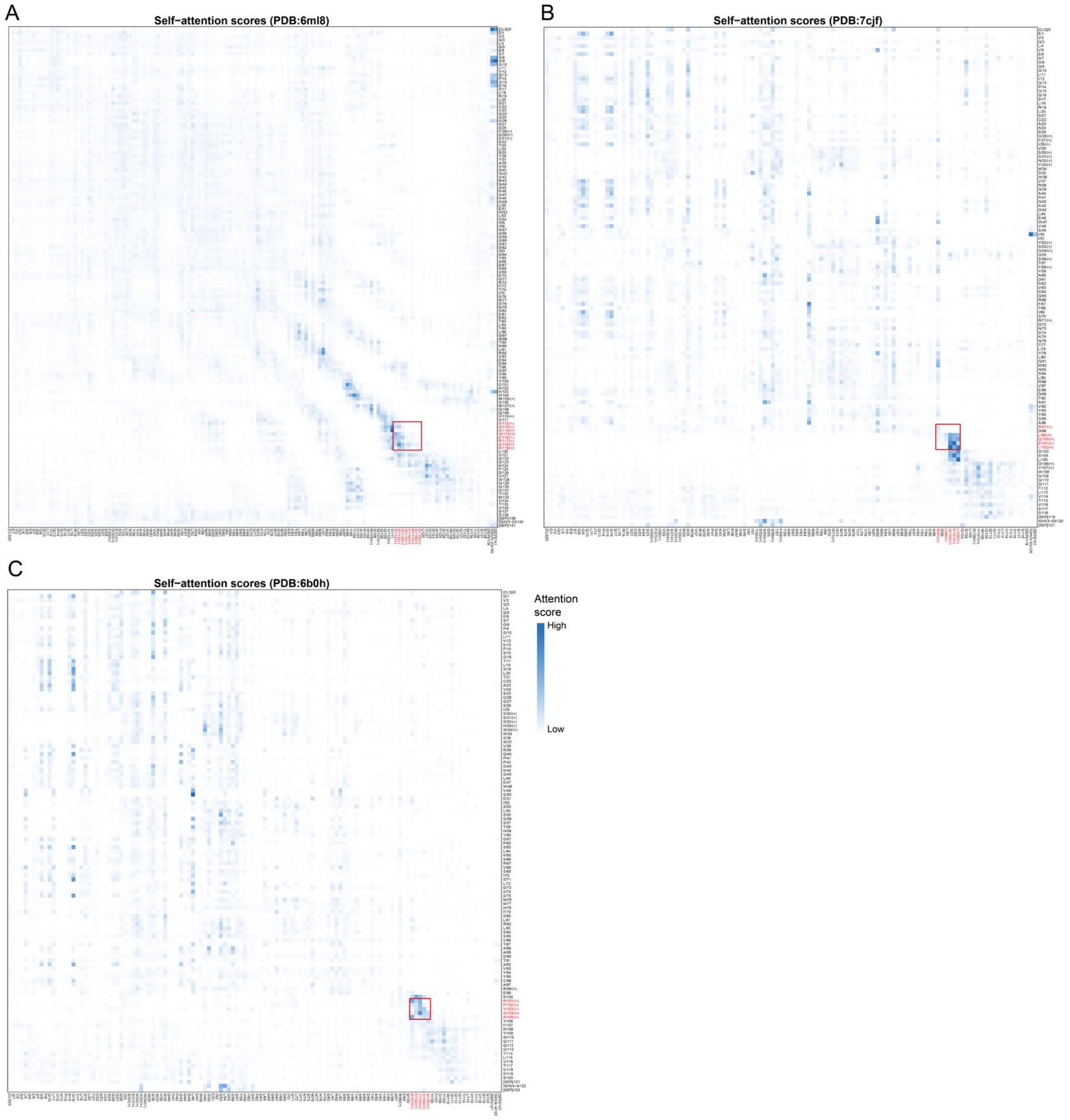

Figure S7. BCRInsight interpretability via self-attention scores. (A-C) Attention heatmaps for PDB structures 6ml8 (A), 7cjf (B), and 6b0h (C). Red boxes indicate high-score regions aligned with CDRs, demonstrating autonomous identification of functional residues and paratopes.

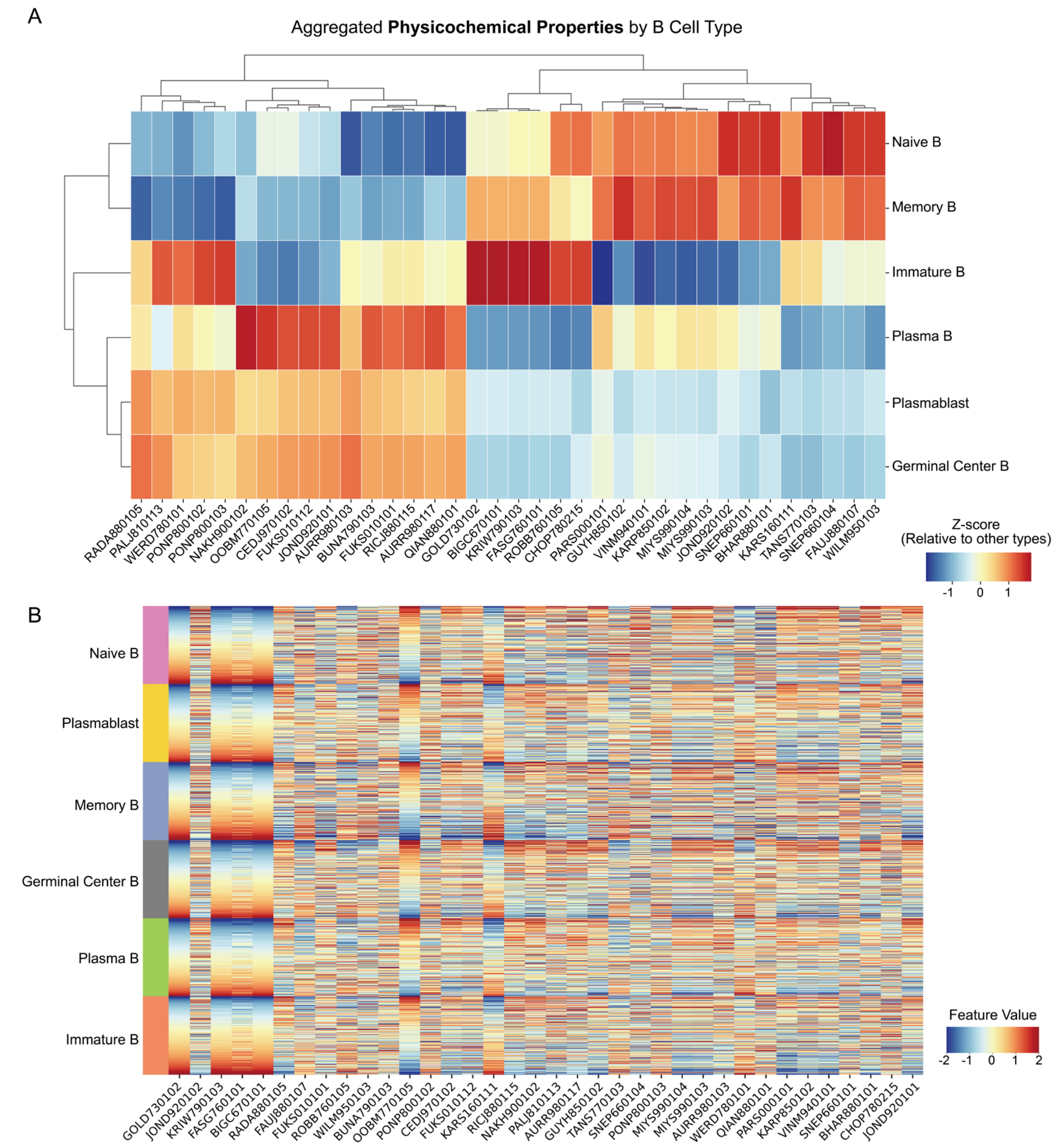

Figure S8. BCR physicochemical landscapes across developmental subsets. (A) Hierarchical clustering of aggregated Z-scores across six stages, showing evolutionary proximity during B-cell differentiation. (B) Feature distribution heatmap. Divergent patterns between subsets (e.g., Naive vs. Plasma B) highlight biophysical shifts during immune maturation.

3. Supplementary Tables

Table S1. Statistical Overview of Training Dataset

Cell type	Immunological condition	BCR count	Sum
Immature B	Healthy	1213602	1,938,945
	First Infection	528920	
	Second Infection	196423	
Naïve B	Healthy/celiac-disease	21289544	52,052,746
	unknown	20189531	
	Healthy	4034664	
	First Infection	2335361	
	MuSK-MG	860806	
	AChR-MG	846962	
	Second Infection	706641	
	Tonsillitis	512378	
	SARS-COV-2	452074	
	Tonsillitis/Obstructive-Sleep-Apnea	331692	
	Obstructive-Sleep-Apnea	249838	
	CMV	243255	
Plasma B	unknown	6714573	7,090,598
	Healthy	250162	
	First Infection	104849	
	Second Infection	21014	
Plasmablast	Tonsillitis	1500070	4,671,191
	unknown	1476119	
	Obstructive-Sleep-Apnea	677088	
	Tonsillitis/Obstructive-Sleep-Apnea	675126	
	Ebola	335479	
	CMV	5040	
	SARS-COV-2	2269	

Memory B	HIV	6502903	18,954,588
	unknown	5749267	
	Tonsillitis	1447010	
	Obstructive-Sleep-Apnea	1239754	
	Tonsillitis/Obstructive-Sleep-Apnea	1082913	
	MuSK-MG	905071	
	AChR-MG	805795	
	Healthy	526943	
	SARS-COV-2	293595	
	First Infection	249468	
	CMV	103959	
	Second Infection	47910	
Germinal Center B	Tonsillitis	2257275	5,168,019
	Obstructive-Sleep-Apnea	1347871	
	Tonsillitis/Obstructive-Sleep-Apnea	1093539	
	unknown	469334	

Table S2. Proportion of antibody types in different cell subsets of the training dataset

Cell Type	Bulk(%)	IGHA(%)	IGHD(%)	IGHE(%)	IGHG(%)	IGHM(%)
GCB[a]	0.598	4.002	1.153	0.001	45.515	48.731
Immature B	0.000	0.007	31.100	0.002	0.413	68.479
Memory B	48.085	6.410	1.435	0.002	14.210	29.857
Naive B	33.471	2.941	10.240	0.002	1.231	52.116
Plasma B	1.614	4.385	1.053	0.006	77.842	15.100
Plasmablast	1.053	11.490	0.020	0.002	79.125	8.309

[a] GCB denotes Germinal Center B-cell.

Table S3. Ablation study on input attributes of B-cell subtype prediction task

Methods	Accuracy(±std)
BCRInsight (HCDR3 & Gene & Isotype)	0.833±0.002
HCDR3	0.691±0.002
HCDR3 & Gene	0.715±0.001
Full length BCR & Gene & Isotype	0.848±0.004

Table S4. Configuration of BCRInsight

BCRInsight	Parameter	Value
Training configures	Batch Size	512
	Number of steps	225000
	Peak learning rate	1e-4
	Learning rate schedule	Plateau-based decay
	Optimizer	AdamW
	Weight decay	0.01
	Warmup ratio	0.05
Model configures	Number of layers	12
	Number of attention heads	12
	Embedding dimension	768
	Feed forward layer dimension	3072
	w_{cl}	0.5

Table S5. Clarification of model feature architecture and actual inference inputs in downstream tasks

Downstream Task	Evaluated Model	Original Model Input Space (Training Features)	Actual Input Features Provided for Inference
UMAP visualization	BCRInsight(HCDR3)/ BCRInsight	HCDR3 or Full-length sequence + V(D)J Gene + isotype	HCDR3 or Full-length sequence + V(D)J Gene + isotype
B-cell subtype prediction	BCRInsight(HCDR3)	HCDR3 sequence + V(D)J Gene + isotype	HCDR3 sequence + V(D)J Gene + isotype
Paratope prediction	BCRInsight	Full-length sequence + V(D)J Gene + isotype	Full-length sequence + V(D)J Gene

Table S6. Key differences between BCRInsight and AntiBERTa/Sapiens

	AntiBERTa	Sapiens	BCRInsight (Our Model)
Objective	Masked Language Modeling (MLM)	MLM	MLM + Contrastive Learning(CL)
Inputs	BCR sequences (mixed heavy and light chains): 42M heavy chains, 15M light chains.	BCR sequences: 20 million unaligned heavy chain amino acid sequences/19 million light chains.	BCR sequences (mixed heavy and light chains): 67M heavy chains + 22M light chains; V(D)J gene tokens; isotype tokens
Label	Labels are derived from the data itself.	Labels are derived from the data itself.	MLM: Labels are derived from the data itself.<br>CL: Labels are derived from annotated B cell type labels.
Sampling	Random sampling	Random sampling	Class-balanced sampling based on B cell types.
Evaluation	Paratope prediction	Humanness: using OASis scores	B-cell subtype prediction + Paratope prediction

Table S7. Harmonization mapping rules and established FACS surface markers for B-cell subtypes.

Subtype	Markers	Biological Hallmarks
Immature	CD45+ CD19+ CD10+ CD27- CD38-	CD10+ is the critical feature distinguishing immature B cells from naive B cells in the periphery.
Naive	CD45+ CD19+ CD10- CD27- CD38-/<br>CD19+ IgD+ CD38-/<br>CD19+ CD27- IgM+ IgD+/<br>CD20+ CD19+ IgD+ CD3- CD27-/<br>CD20+ CD27-	Typical double- or triple-negative features (e.g., CD27- CD38-), often marked by IgD+/IgM+ co-expression.
Memory	CD45+ CD19+ CD10- CD27+ CD38-/<br>CD19+ CD27+ IgM+ IgD-/<br>CD19+ CD27+ IgM- IgD-/<br>CD20+ CD19+ IgD- CD3- CD27+/<br>CD20+ CD27+	CD27+ serves as the classic universal marker for memory B cell subsets.
Plasma	CD45+ CD19+ CD10- CD27+ CD38+/<br>CD19+ CD20- CD27+ CD38+ HLA-DR+/<br>CD19+ CD20- CD27+ CD38+ HLA-DR-	Characterized by the loss of CD20 and exceptionally high expression of CD38+.
Plasmablast	CD45+ CD19+ CD19+ IgD- CD38+/<br>CD19+ CD3- IgDlow CD20low CD38+ CD71+ BLIMP1+/<br>CD19+ IgD- CD71hi CD38hi CD20-	Characterized by active proliferation markers (CD71+) and transition phenotypes.
Germinal Center B	CD45+ CD19+ CD19+ IgD- CD38+/<br>CD19+ CD3- IgDlow BCL6+ CD38int	Driven by the master transcription factor BCL6+; distinct from naive and memory pools.
